# Therapeutic Potential of CUDC-907 (Fimepinostat) for Hepatocarcinoma Treatment Revealed by Tumor Spheroids-Based Drug Screening

**DOI:** 10.3389/fphar.2021.658197

**Published:** 2021-10-29

**Authors:** Wei Liao, Wanren Yang, Jiecheng Xu, Zhengming Yan, Mingxin Pan, Xiaoping Xu, Shuqin Zhou, Yu Zhu, Jianqiang Lan, Min Zeng, Xu Han, Shao Li, Yang Li, Kangyan Liang, Yi Gao, Qing Peng

**Affiliations:** ^1^ General Surgery Center, Department of Hepatobiliary Surgery II, Guangdong Provincial Research Center for Artificial Organ and Tissue Engineering, Guangzhou Clinical Research and Transformation Center for Artificial Liver, Institute of Regenerative Medicine, Zhujiang Hospital, Southern Medical University, Guangzhou, China; ^2^ Department of Anesthesiology, Zhujiang Hospital, Southern Medical University, Guangzhou, China; ^3^ Accurate International Biotechnology Co., Guangzhou, China; ^4^ State Key Laboratory of Organ Failure Research, Southern Medical University, Guangzhou, China

**Keywords:** three-dimensional culture, drug screening, hepatocarcinoma, primary cells, CUDC-907 (fimepinostat)

## Abstract

**Background:** Cancer is the second leading cause of death globally. However, most of the new anti-cancer agents screened by traditional drug screening methods fail in the clinic because of lack of efficacy. Choosing an appropriate *in vitro* tumor model is crucial for preclinical drug screening. In this study, we screened anti-hepatocarcinoma (HCC) drugs using a novel spheroid cell culture device.

**Methods:** Four HCC cell lines were three-dimensionally (3D) cultured to screen 19 small molecular agents. 3D-cultured primary HCC cells and a tumor-bearing mouse model were used to verify the candidate anti-hepatocarcinoma agent. Cell function experiments and western blotting were conducted to explore the anti-hepatocarcinoma mechanism of the candidate agent.

**Results:** We found that CUDC-907 can serve as a potent anti-hepatocarcinoma agent. The study data show that CUDC-907 (fimepinostat), a novel dual acting inhibitor of phosphoinositide 3-kinase (PI3K) and histone deacetylase (HDAC), has potent inhibitory effects on HCC cell lines and primary HCC cells *in vitro*, Animal studies have shown that CUDC-907 can also suppress HCC cells *in vivo*. Furthermore, we found that CUDC-907 inhibits the PI3K/AKT/mTOR pathway and downregulates the expression of c-Myc, leading to the suppression of HCC cells.

**Conclusion:** Our results suggest that CUDC-907 can be a candidate anti-HCC drug, and the 3D *in vitro* drug screening method based on our novel spheroid culture device is promising for future drug screening efforts.

## Introduction

Globally, approximately one in six deaths are attributed to cancer, making it the second leading cause of death (Bray et al., 2018). The great progress in molecular targeted therapy has successfully prolonged the survival time of cancer patients (Lee et al., 2018). However, many refractory cancers, such as hepatocellular carcinoma (HCC), still lack potent anti-tumor molecular agents (Lee et al., 2021). Therefore, substantial efforts are still needed to discover and screen new anti-cancer molecular agents for HCC treatment.

Developing new drugs is a very expensive process. One of the main reasons for the high cost of drug development is the high risk of clinical failure of new drugs. Thus, choosing an effective preclinical model that can improve the predictive power in the drug screening process is an impactful measure to reduce the cost of drug development and greatly promote the successful treatment of cancer patients.

*In vitro* cancer models, mainly two-dimensional (2D) and three-dimensional (3D) cancer models, are frequently used for drug screening. Compared with the 2D cancer model, the 3D cancer model, which can mimic the *in vivo* cancer microenvironment and recapture the original cancer characteristics, shows great potential for *in vitro* drug screening (Cheng et al., 2013; Ryu et al., 2019). Besides, primary cancer cells obtained from patients could maintain their primitive genetic background and features for a long time (Song et al., 2018). Therefore, the combination of 3D culture and primary cancer cells may improve the accuracy of anti-tumor drug screening.

HCC is one of the most lethal malignancies worldwide, but lacks potent molecular targeted anti-HCC agents (Llovet et al., 2018; Li et al., 2020). Considering that cell cycle interruption and DNA damage induction are the main mechanism for tumor suppression (Matthews et al., 2021), we chose 19 molecular inhibitors, which target cell cycle or DNA damage and have been rarely studied in HCC, for this study. Using an *in vitro* 3D drug screening method on our spheroid culture system, we found that the molecular inhibitor CUDC-907 could serve as a potential anti-HCC agent. CUDC-907, also known as Fimepinostat, is a synthetic and orally available small molecular agent. It inhibits both phosphatidylinositol 3-kinase (PI3K) and histone deacetylase (HDAC) (Qian et al., 2012). At present, CUDC-907 has not yet been approved by the FDA as a treatment option for any disease and is still undergoing clinical trials on some types of cancers (Younes et al., 2016a; Oki et al., 2017). In preclinical studies, CUDC-907 has been proposed to have therapeutic potential in many hematopoietic malignancies and solid cancers, such as chronic lymphocytic leukemia, acute myeloid leukemia, thyroid cancer, and pancreatic adenocarcinoma (Kotian et al., 2017; Chen et al., 2019a; Fu et al., 2019; Li et al., 2019; Qiao et al., 2021). In this study, we investigated the anti-tumor effect of CUDC-907 on HCC cell lines *in vitro* and *in vivo*. Primary HCC cells were used to verify the efficacy of CUDC-907 using a 3D method.

## Materials and Methods

### Fabrication of Agarose Microwells in a 96-Well Plate

The method for fabrication of agarose microwells in a 96-well plate was described in detail in a previous study (Liao et al., 2019). Briefly: 1) the stamp-like molds were designed and created using Solidwork software (Dassault Systems, Concord, United States). 2) The resin molds were printed using a Formlabs Phoenix Touch Pro UV-LED 3D printing system (Full Spectrum Laser, Las Vegas, NV, United States). 3) 2% (w/v, agarose/deionized water) melted agarose (111860, BioWest Regular, Hong Kong, China) was added to 96-well plates (Black Plate, Clear Bottom 96 Well Assay plate, 3603, Corning, NY, United States) (170 μl/well). 4) Sterilized mold was used to shape the liquid agarose and pull out the mold when the agarose was cool. 5) Before using the agarose microwells, 170 μl of Dulbecco’s Modified Eagle’s Medium (C11995500BT, DMEM, Gibco, Beijing, China) was added to saturate each agarose well. After 15 min, the culture medium was removed, and the process was repeated three times. The agarose concave 96-well culture plate was then ready for use.

### HCC Cell Line Culture and Spheroid Formation

The human HCC cell lines HuH-7, BEL-7402, and SMMC-7721 were purchased from the Cell Bank of the Chinese Academy of Sciences, Shanghai, China. SNU-449 cells were purchased from ATCC (American Type Culture Collection). All cell lines were cultured in DMEM with 10% fetal bovine serum (A3160801, Gibco). For spheroid formation, cells were seeded onto a novel spheroid culture plate at a density of 1,500 cells/well. HCC cells were then cultured in DMEM with 10% fetal bovine serum and incubated at 37°C in a humidified atmosphere with 5% CO_2_.

### Drug Screening of HCC Cell Lines in Spheroid Culture

The HCC cell lines were incubated for 2 days in 3D culture plates (1,500 cell/well) and the drug sensitivity test was then performed. In the first round of drug screening, 19 molecular inhibitors targeting the cell cycle and DNA damage were selected from an inhibitor screening library (HYCPK1971, MedchemExpress, NJ, United States). Specifically, inhibitors were diluted to 10 µM with culture medium and then added into the spheroid culture well after 2–3 days of culture. After 3 days of treatment, we used the CellTiter-Glo® 3D Cell Viability Assay (G9683, Promega Corporation, Madison, United States) to measure the fluorescence intensity (representing cell viability) of each culture well, according to the manufacturer’s instructions. Then, we calculated the inhibition ratio of each inhibitor: inhibition ratio = (Fluorescence intensity of control group−Fluorescence intensity of experimental group)/(Fluorescence intensity of control group−Fluorescence intensity of blank comparison group). Inhibitors with inhibition ratios above 85% were selected. After that, the second round of drug screening was executed. The inhibitors identified by the first-round screening were serially diluted to six concentrations with the culture medium, and the highest concentration was 10 µM. The drugs were added into the 3D culture wells and cells were treated for 3 days. Then, the cell activity of HCC spheroids and the inhibition ratio were measured and calculated. Finally, the inhibitor with the best inhibitory effect on HCC cells was chosen and the last round of drug screening was conducted. Four HCC cell lines were cultured in our novel spheroid culture plate, and these cell spheroids were treated with the last selected inhibitor, serially diluted to six concentrations with culture medium for 3 days. Cell activity was measured and the inhibitory concentration of 50% (IC_50_) of this inhibitor on four different cells was calculated by using the Prohibit method with SPSS 20.0 software. Each assay was performed in triplicate.

### Isolation, Culture, and Drug Sensitivity Test of Human Primary HCC and Hepatic Cells

The HCC and liver samples were obtained with the consent of the patients and the hospital ethics committee (approval document number: 2017-GDEK-004). The isolation and culture method of primary HCC cells has been described previously (Liao et al., 2019). In short, the isolation and culture processes are as follows: immediately after surgery, specimens were obtained and transported to the laboratory at 0°C in DMEM. The specimens were rinsed 2–3 times with DMEM to remove the blood and were cut into small fragments (1 mm^3^ or smaller). The cut specimens were placed in Hank’s Balanced Salt Solution (HBSS, 14025076, Gibco) containing 0.1% type IV collagenase (17104-019, Gibco) and digested for 40–90 min at 37°C. The resulting suspension was filtered through a 100-μm nylon filter and centrifuged at 50 × g for 3 min at 4°C. Then, the pellet was washed twice with HBSS. The final cell suspensions were cultured in T25 flasks (TCF001050, BIOFIL, Guangzhou, China) and hepatocyte culture medium (CC-3198, Lonza, Basel, Switzerland) at 37°C in a humidified incubator with 5% CO_2_. The medium was changed at 24 h after seeding to remove dead cells and debris.

For the isolation of normal hepatocytes, the method differed from the isolation of HCC cells. Specifically, 0.1% type IV collagenase solution was warmed by thermostat water bath and kept at 37°C. Then, the digest solution was slowly and continually injected into different parts of liver tissue through disposable sterile injectors for about 30 min. The tissue was cut into pieces and rinsed in the digest solution until the whole tissue was fully digested and had become soft. After that, the digest solution was collected and filtered through a 100-μm nylon filter. The remaining isolation and culture steps were the same as those used for culturing primary HCC cells. After 2–3 days of culture, the primary cells were harvested and seeded onto our novel spheroid culture plates at a density of 1,500 cells/well for drug sensitivity testing. The method of drug-sensitivity testing of CUDC-907 on primary cells is as mentioned above.

### Animal Studies

Male Balb/c nude mice (4–5-weeks-old) were purchased from the Animal Experimental Center of Southern Medical University. The animal study was carried out in strict accordance with the Guidelines for the Care and Use of Laboratory Animals, China. All experimental protocols involving animals were approved by the Laboratory Animal Ethics Committee of Zhujiang Hospital of Southern Medical University (approval document number: LAEC-2020-156). The SMMC-7721 cells at a density of 5 × 10^6^ in 0.2 ml of PBS were inoculated subcutaneously into the left flank of the nude mice. When the tumor volume reached approximately 100 mm^3^ at approximately 1 week, the mice were randomly assigned to the vehicle control (*n* = 5) and treated groups (*n* = 5), and they were treated with 30% propylene glycol or 300 mg/kg CUDC-907 by intragastric administration, three times in 1 week, with 1- or 2-days intervals, respectively. The tumors and body weights of the mice were measured individually twice weekly. The volume of the tumor was calculated using the following formula: volume = length × width × height × π/6. After treatment for 18 days, the mice were sacrificed after the final therapy. Tumors were removed and lysed for western blotting or fixed for immunohistochemistry.

### Calcein-AM/Propidium Iodide Staining

The cells were incubated in phosphate-buffered saline (PBS, C10010500BT, PBS, Gibco) with 2 μM calcein-AM and 2.5 μg/ml PI (C326 and P346, Dojindo Chemical Technology, Shanghai, China) for 10 min at room temperature, and then the spheroids were examined and photographed under a fluorescence microscope.

### Flow Cytometry–Apoptosis

SMMC-7721 and Huh-7 cells were treated with CUDC-907 of different concentrations for 36 h. To detect apoptosis, the Annexin V FITC Apoptosis Detection Kit (AD10, Dojindo) was used. Cells were stained with annexin V and propidium iodide (PI) for 30 min at room temperature in the dark, according to the manufacturer’s instructions. Cells were kept on ice before analysis by flow cytometry (FACSVerse, BD Biosciences, CA, United States).

### Flow Cytometry–Cell Cycle

SMMC-7721 and Huh-7 cells were treated with CUDC-907 of different concentrations for 24 h. After the treatment, cells were washed with PBS, harvested, centrifuged two times at 1,200 rpm at 4°C for 5 min, and resuspended in 300 μl of sodium citrate buffer containing 0.1% Triton X-100, 100 μg/ml RNase, and 1 mg/ml DAPI. The cell cycle distribution was determined by flow cytometry analysis using a flow cytometer (FACSVerse, BD Biosciences). Each assay was repeated in triplicate, and the data were analyzed using ModfitLT (Verity Software House, ME, United States).

### Western Blotting

Cells were pelleted by centrifugation, washed once with ice-cold PBS, and lysed on ice for 30 min using the cell lysis buffer (p0013, Beyotime, Shanghai, China) according to the manufacturer’s extraction protocol. Protein quantitation was performed using the BCA Protein Assay Kit (ab102536, Abcam, Shanghai, China). A total of 30 μg of protein was denatured in Laemli buffer at 95°C for 5 min, and western blotting was performed using the Bio-Rad system (TGX 10–15% gels). Transfer was performed using the Trans Blot turbo system (Bio-Rad) into PVDF membranes. Images were acquired using the Bio-Rad Imaging Chemidoc XPS + system. Secondary anti-rabbit and anti-mouse HRP-conjugated antibodies were purchased from Beyotime (A0208; A0216). Proteins were detected using the following antibodies: PARP1 (66520-1-Ig, Proteintech, Wuhan, China); Caspase 3 (9662, Cell signaling technology, Shanghai, China); P21 (60214-1-Ig, Proteintech); Cyclin D1 (60186-1-Ig, Proteintech); AKT (60203-2-Ig, Proteintech); AKT-phospho-S473 (66444-1-Ig, Proteintech); 4EBP1 (60246-1-Ig, Proteintech); c-MYC (10828-1-AP, Proteintech); Histone H3 (acetyl K9) (ab32129, Abcam); S6 Ribosomal Protein (AF6354, Affinity, Jiangsu, China); Phospho-S6 Ribosomal Protein (Ser235/236) (4858T, Shanghai, China), and Phospho-4E-BP1 (S65) (YP0618, Immunoway, TX, United States).

### Immunofluorescence Analysis

To identify the extracted primary cells, immunofluorescence analysis was performed. The primary cells were fixed with 4% paraformaldehyde for 10 min at room temperature, permeabilized with 0.1% Triton X-100 for 20 min at room temperature, and then incubated with 5% BSA and 0.5% Triton in TBS solution for 1 h. The following primary antibodies were purchased from Abcam: anti-human serum albumin (ALB, ab207327), anti-human keratin 18 (CK-18, ab668), anti-human Arginase1 (ARG-1, ab212522), and anti-human alpha fetoprotein (AFP, ab169552). The samples were incubated with primary antibodies for 16 h at 4°C. The secondary antibodies were anti-mouse IgG conjugated with Alexa® Fluor 488 (Abcam, ab 150078) and goat anti-rabbit IgG conjugated with Alexa® Fluor 555 (Abcam, ab150117). The samples were incubated with secondary antibodies for 1 h and incubated with DAPI (Abcam) for 10 min at room temperature in the dark. All fluorescent images were obtained using a Leica fluorescence microscope.

### Immunohistochemistry Staining

The xenograft tumor tissues were fixed with 4% formalin and cut into 4 µm thin sections. For immunohistochemical staining, paraffin slides were deparaffinized and subjected to antigen retrieval using citrate sodium solution (pH 6.0). The slides were then incubated in TBS solution containing 1% BSA and 0.5% Triton for 1 h at room temperature. Slides were incubated with primary antibodies for Ki67 (ab15580, Abcam) and c-Myc (10828-1-AP, Proteintech) at 1:500 dilution overnight at 4°C. Endogenous peroxidase activity was blocked in 3% hydrogen peroxide/methanol buffer for 15 min at room temperature. Detection of bound antibodies was accomplished with the Streptavidin-Peroxidase kit (sp-9001, ZSGB-BIO, Beijing, China). Immunohistochemistry was independently assessed using the quick score method. Each specimen was assigned a score according to the intensity of the nucleic staining (no staining = 0; weak staining = 1; moderate staining = 2; and strong staining = 3), and the proportion of positive cells was estimated and given a percentage score on a scale from 1 to 6 (1–4% = 1; 5–19% = 2; 20–39% = 3; 40–59% = 4; 60–79% = 5; and 80–100% = 6). The final immune-reactive score was determined by multiplying the intensity score by the extent of score of stained cells, ranging from a minimum of 0 to a maximum of 18.

### TdT-Mediated dUTP Nick-End Labeling Assay

The xenograft tumor tissues were fixed with 4% formalin and cut into 4 µm thin sections. For the TUNEL assay, paraffin slides were deparaffinized and washed with PBS. The TUNEL assay was then performed using the Colorimetric TUNEL Apoptosis Assay Kit (C1091, Beyotime), following the manufacturer’s instructions. The percentage of apoptotic area in tumor slides in TUNEL staining was calculated by IMAGE J software.

### Polymerase Chain Reaction and Sanger Sequencing

PCR and Sanger Sequencing were conducted with the assistance of Regene Biotech (Guangzhou, China). In short, Genomic DNA was extracted from HCC sample with the genomic DNA preparation kit (D3121, Magen Biotechnology). The polymerase chain reaction (PCR) of PI3K gene was performed using the extracted genomic DNA with designed primers and PCR master mix (P213-01, Vazyme Biotech) in S1000™ thermal cyclers (BIO-RAD, United States), according to the manufacturer’s instructions. The designed sequences of primers for PI3K mutation sites were shown in Table 1.

**TABLE 1:** The designed sequences of primers for PI3K mutation sites.

Mutation site	Forward primer	Reverse primer
rs121913279	TCA​GGA​GAT​GTG​TTA​CAA​GG	GAA​TCC​AGA​GTG​AGC​TTT​CA
rs121913273/rs121913275/rs104886003	TTG​GTT​CTT​TCC​TGT​CTC​TG	ATG​TGC​CAA​CTA​CCA​ATG​TA

For direct mutation screening, automated DNA Sequencer (ABI Genetic Analyzer) following Sanger’s dideoxy chain termination method was used. Sequence alignment report was generated by chromas software and online BLAST sequence analyzer.

### Statistical Analysis

All experiments were performed at least three times. The results are presented as mean ± SD. Statistical analysis was performed using two-tailed unpaired Student’s t-tests. *p* < 0.05 was considered statistically significant. The univariate linear regression analysis was calculated with SPSS 20.0 software.

## Results

### CUDC-907 has a Potent Inhibitory Effect on Proliferation of HCC Cell Lines in Spheroid-Based Drug Screening Experiments

**FIGURE 1:**
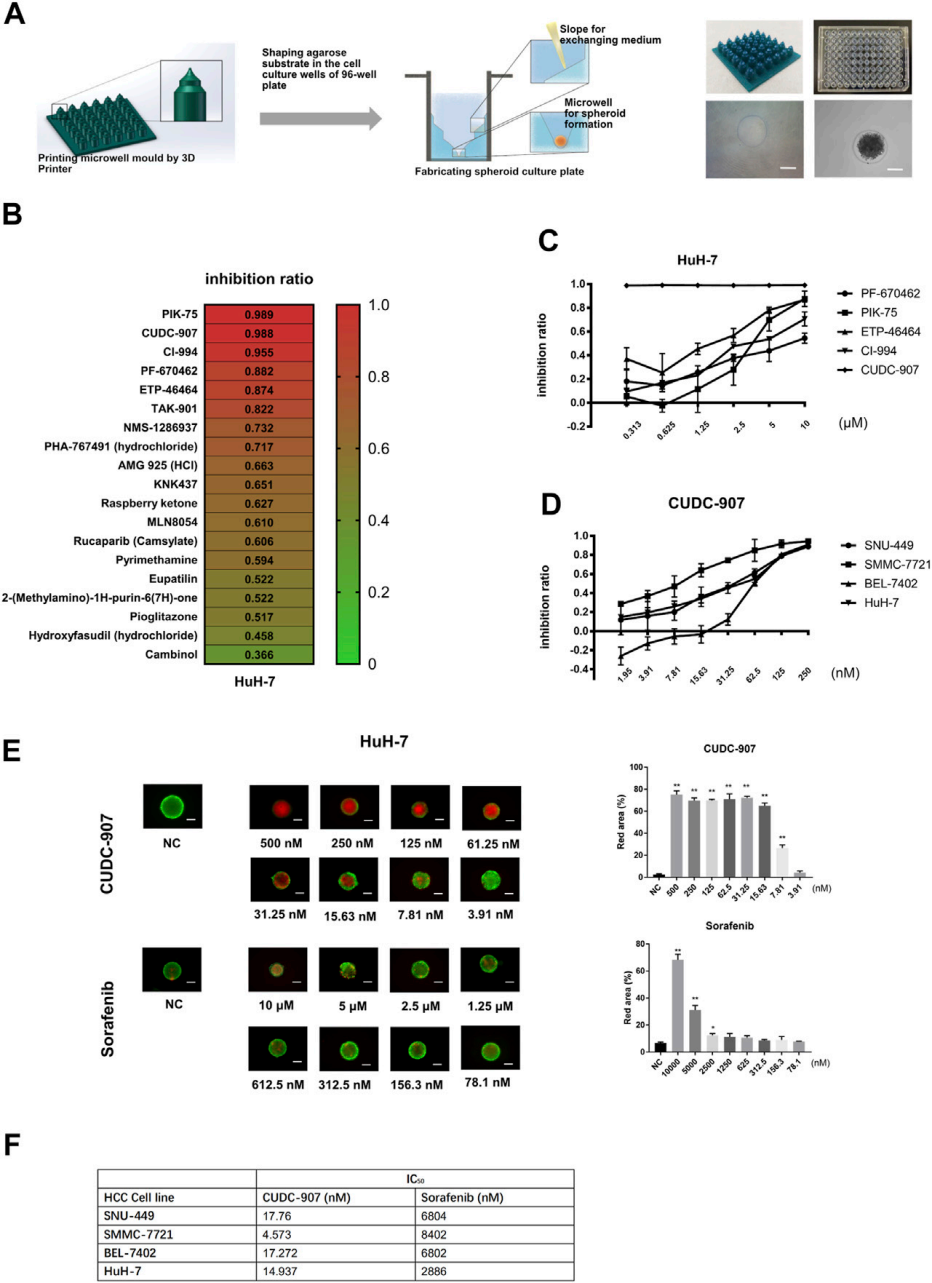
CUDC-907 was screened as a potential anti-HCC agent by *in vitro* 3D drug screening. **(A)** The method for fabrication of agarose microwells in a 96-well plate. 3D-printed resin mold and the ameliorated 96-well plate with shaped agarose substrate are shown. **(B)** The first round of drug screening. HuH-7 cells were cultured in our novel 3D-culture plate and were treated with 19 inhibitors at a concentration of 10 μM for 3 days. **(C–D)** Inhibitory curve of different inhibitors on HCC cell lines. HCC cell lines were cultured in our novel spheroid culture device and treated with different agents for 3 days. **(E)** Calcein-AM/propidium iodide staining of HuH-7 spheroids. The HuH-7 cells were treated with different concentration of CUDC-907 and sorafenib for 3 days. **(F)** The IC_50_ of sorafenib and CUCU-907 on four HCC cell lines. Scale bar = 200 μM. *p < 0.05, **p < 0.001 (result compares with NC group).

In order to provide a cheaper, stable, and standardized tool for *in vitro* 3D drug screening, we developed a novel stamp-like resin mold that can shape the agarose to form a special 3D structure for spheroid culture (Figure 1A) and designed a drug screening process based on this spheroid culture system. HCC cells could form uniform spheroids in our novel 3D culture system (Figure 1A). Using this 3D culture system, we screened 19 molecular inhibitors targeting the cell cycle and DNA damage using the HuH-7 cells, and screened out five potent agents (PF-670462, PIK-75, ETP-46464, CI-994, and CUDC-907) with inhibition ratios above 85% at 10 µM (Figure 1B). We then tested the inhibitory ratios of the above five agents with different concentrations on the HuH-7 cell line (Figure 1C) and calculated the IC_50_ of each agent. The results showed that the inhibitory potency of CUDC-907 was stronger than that of the other four agents. We then used four HCC cells (SMMC-7721, HuH-7, SNU-449, and BEL-7402) to examine the inhibitory efficacy of CUDC-907 through 3D method and compare its effect with sorafenib. We found that the IC_50_ of CUDC-907 on HCC lines ranged from 4.573 to 17.76 nM, which was far lower than that of sorafenib (Figures 1D,F, Supplementary Figure S1A). Afterward, we used calcein-AM/propidium iodide staining to visualize the inhibitory effect of CUDC-907 and sorafenib. The data showed that CUDC-907 was able to kill HuH-7 cells at a low concentration and its killing effect was much stronger than that of sorafenib (Figure 1E).

### CUDC-907 Inhibits Proliferation of Primary HCC Cells and the *In Vitro* Inhibitory Efficacy is Stronger Than That of Sorafenib

**FIGURE 2:**
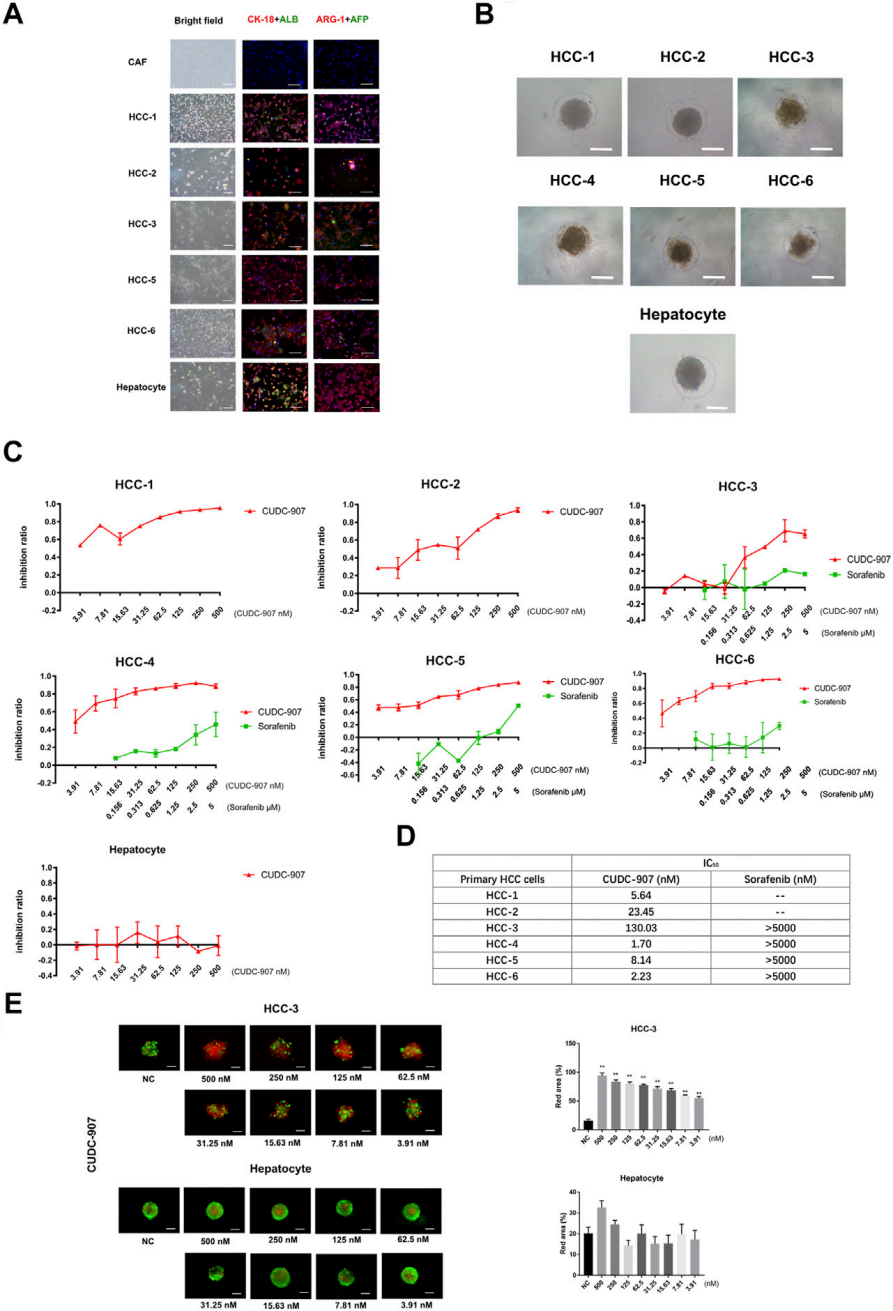
The drug sensitivity test of primary cell spheroid. **(A)** AFP, albumin, ARG-1 and CK-18 immunostaining of primary cells to examine the cellular origin of primary cells. **(B)** Primary cells cultured in our novel 3D culture device for 3 days. **(C)** Inhibitory curve of CUDC-907 and sorafenib on primary cell spheroid. Primary cell spheroids were treated with CUDC-907 and sorafenib for 3 days. **(D)** The IC_50_ of CUDC-907 and sorafenib on primary HCC spheroids. **(E)** Calcein-AM/propidium iodide staining of primary cell spheroids. The primary cells were treated with CUDC-907 for 3 days. Scale bar = 200 μM. *p < 0.05, **p < 0.001 (result compares with NC group).

To provide convincing evidence for the inhibitory efficacy of CUDC-907 on HCC cells, we used primary cells to verify the effect of CUDC-907. In this experiment, we successfully extracted and cultured primary HCC cells from six HCC patients (Table 2) and primary hepatocytes from the adjacent normal liver tissue of a sarcomatoid hepatocellular carcinoma patient. HCC cells from 5 to 6 patients and the primary hepatocytes were stained with CK-18, ALB, ARG-1 (biomarkers of hepatocyte and hepatocarcinoma cells) and AFP (biomarker of hepatocarcinoma cells), with cancer-associated fibroblasts (CAFs, from HCC sample) as the negative control. The results showed that most of the cells extracted from cancer tissue were HCC cells, and hepatocytes highly expressed CK-18, ALB, and ARG-1 (Figure 2A). Then, we cultured these primary cells in our novel 3D culture plate, and the results showed that the cells formed uniform and regular spheroids after 48 h (Figure 2B). After primary cells formed regular spheroids, 3D-cultured primary cells from seven cases were treated with CUDC-907 for 3 days, and primary HCC spheroids from 4 to 6 cases were treated with sorafenib (the first-line anti-HCC drug) for 3 days for comparison. The inhibition curves showed that CUDC-907 has a high inhibitory efficacy on primary HCC cells, and its efficacy is stronger than that of sorafenib; however, the inhibitory effect of CUDC-907 on primary hepatocytes was much lower, even with the highest drug concentration (Figure 2C). The IC_50_ of CUDC-907 of six cases of primary HCC cells were different, ranging from 1.7 to 130.03 nM, which were far lower than the IC_50_ of sorafenib (>5,000 nM, Figure 2D). In addition, the calcein-AM/propidium iodide staining experiment revealed that CUDC-907 concentrations from 3.91 to 500 nM were able to kill primary HCC cells from HCC-3 patients, although the killing ability of CUDC-907 on primary hepatocytes was not apparent (Figure 2E).

**TABLE 2:** Donor characteristics at the time of HCC resection.

Patient characteristics	HCC-1	HCC-2	HCC-3	HCC-4	HCC-5	HCC-6
Age/sex	69/male	27/female	64/male	51/male	69/male	52/male
HBsAg	Positive	Positive	Positive	Positive	Positive	Positive
Serum HBV DNA	3.05 × 10^5^ IU/ml	7.08 × 10^2^ IU/ml	1.08 × 10^4^ IU/ml	4.36 × 10^5^ IU/ml	—	—
Anti-HCV antibody	Negative	Negative	Negative	Negative	Negative	Negative
Serum α-fetoprotein	4 μg/L	26.8 μg/L	144.2 μg/L	2,590 μg/L	2.9 μg/L	2.8 μg/L
HCC pathologic diagnosis	Highly differentiated hepatocellular carcinoma	Poorly differentiated hepatocellular carcinoma	Moderately differentiated hepatocellular carcinoma	Highly differentiated hepatocellular carcinoma	Moderately differentiated hepatocellular carcinoma	Moderately differentiated hepatocellular carcinoma

### CUDC-907 Inhibits the Growth of HCC Xenografts *In Vivo*

**FIGURE 3:**
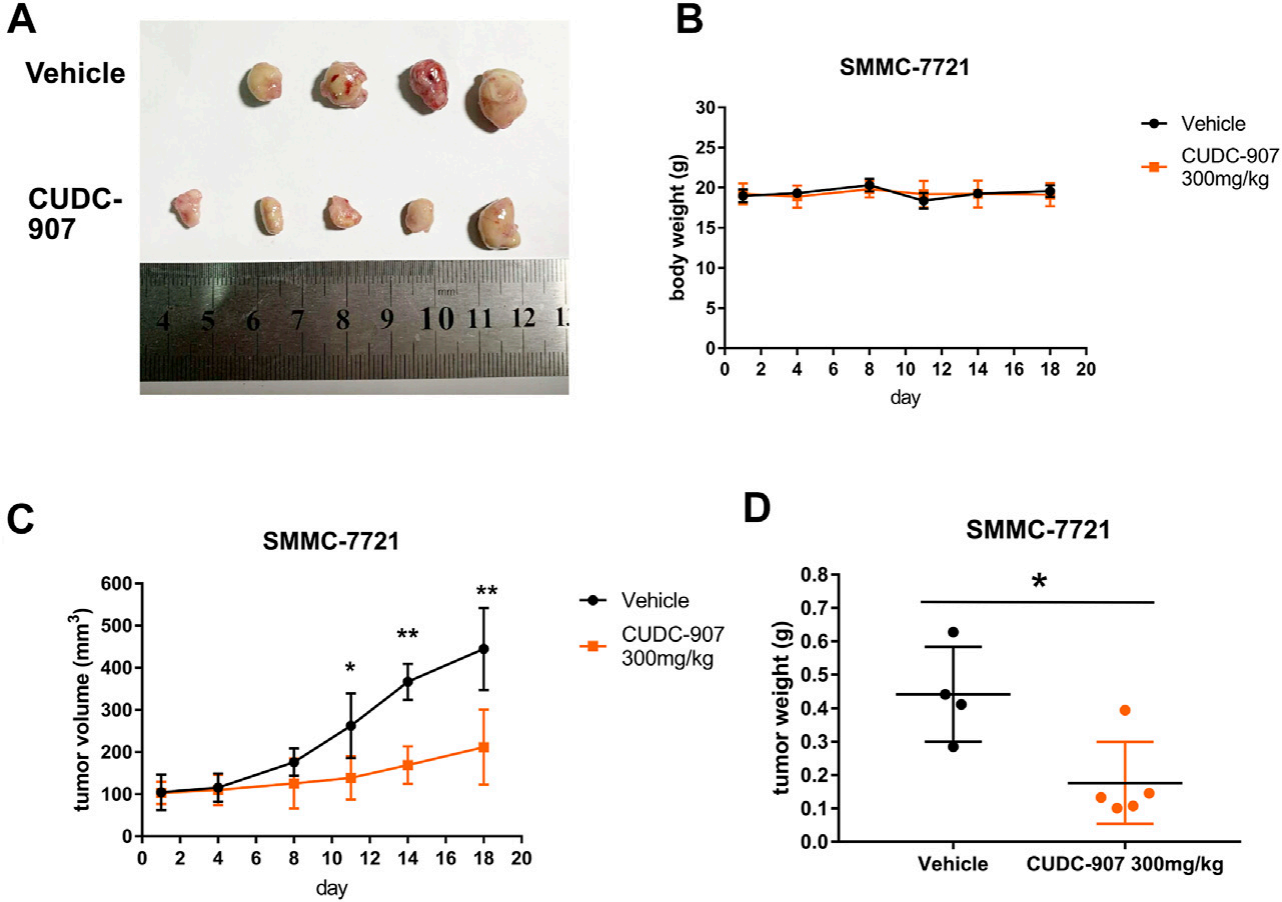
CUDC-907 inhibits HCC cancer growth *in vivo*. **(A)** Tumor morphology. After 18 days, all nude mice were sacrificed, and the tumors were resected to be imaged. **(B–C)** Body weight and tumor volume curves for 18 days of treatment with CUDC-907 or vehicle. **(D)** Tumor weight after 18 days of treatment. *p < 0.05, **p < 0.001 (result compared with Vehicle group).

We next established a xenograft HCC model to evaluate the antitumor effect of CUDC-907 *in vivo*. SMMC-7721 xenograft-bearing mice were treated with 300 mg/kg CUDC-907 three times a week. After the final treatment on day 18, all the nude mice were sacrificed, and tumors were resected, imaged, measured, and weighed as shown in Figure 3. Figure 3A shows that there were only four tumors resected from the untreated mice because the tumor from one of the treated mice grew too large and ruptured. Therefore, the data from this mouse were excluded. The results of animal experiments showed that CUDC-907 treatment significantly reduced tumor growth in the xenograft HCC model compared to the vehicle group (Figures 3C,D), and the body weight of mice showed no significant reduction (Figure 3B). Taken together, these findings suggest that CUDC-907 has a potential therapeutic effect on HCC xenografts.

### CUDC-907 Inhibits Proliferation, Causes G2/M Arrest, and Induces Apoptosis in HCC Cells

**FIGURE 4:**
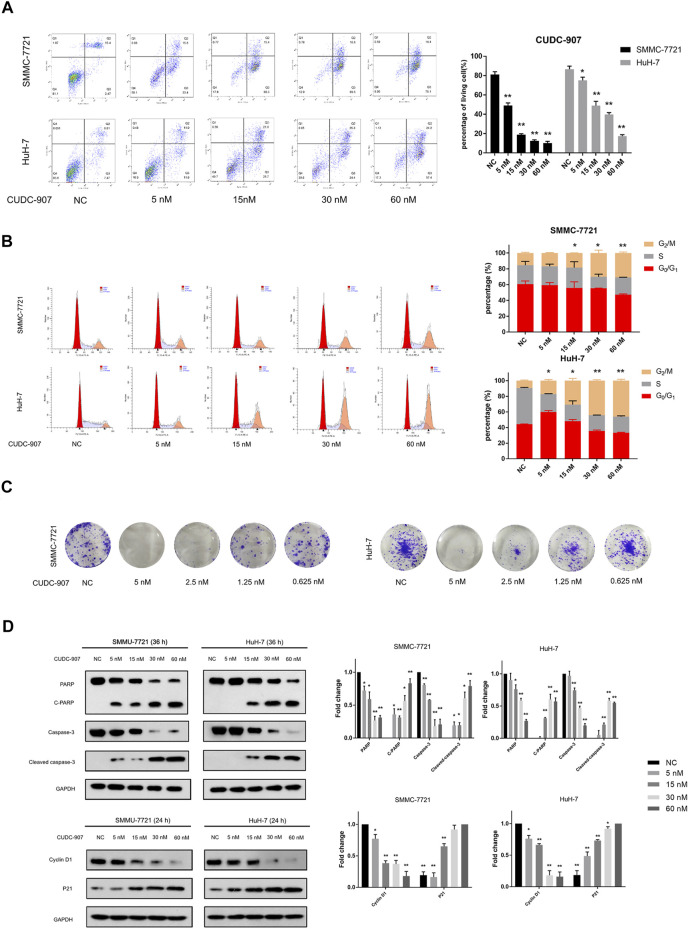
CUDC-907 inhibits proliferation, causes G2/M arrest, and induces apoptosis in HCC cells. **(A)** Cell apoptosis detected by flow cytometry. HuH-7 and SMMC-7721 cells were treated with 0, 5, 15, 30, and 60 nM CUDC-907 for 36 h. **(B)** Cell cycle changes detected by flow cytometry. HuH-7 and SMMC-7721 cells were treated with 0, 5, 15, 30, and 60 nM CUDC-907 for 24 h. The statistic is referring to G2 cell phase. **(C)** Colony-formation assays. HuH-7 and SMMC-7721 cells were measured with crystal violet staining after treatment with CUDC-907 at 0.625–5 nM for 14 days. **(D)** HuH-7 and SMMC-7721 cells were collected for western blotting with the indicated antibodies after treatment with CUDC-907 for 24 h or 36 h. *p < 0.05, **p < 0.001. (C-PARP and Cleaved caspase-3 were compared with PARP or caspase-3 of NC group; P21 was compared with that of 60 nM group; other results were compared with NC group).

Flow cytometry was used to evaluate changes in the cell cycle and apoptosis of HCC cells. SMMC-7721 and HuH-7 cells were treated with 5, 15, 30, and 60 nM CUDC-907 for 24 h for cell cycle assessment and 36 h for apoptosis assessment. CUDC-907 arrested SMMC-7721 and HuH-7 cells at the G2/M phase and strongly induced cell apoptosis in a concentration-dependent manner (Figures 4A,B). Moreover, the colony assay validated the inhibitory effect of CUDC-907 on HCC cell lines (Figure 4C). Furthermore, key factors involved in cell apoptosis and cell cycle arrest were analyzed by western blotting. CUDC-907 markedly upregulated levels of p21 (CDKN1A), cleaved poly (ADP-ribose) polymerase, and Cleaved caspase-3, which were accompanied by decreases in the levels of the corresponding precursors, and downregulated Cyclin D1 expression (Figure 4D). These results suggested that CUDC-907 may inhibit HCC cell proliferation in part through cell cycle arrest and/or induction of cell apoptosis.

### CUDC-907 Inhibits HDAC and PI3K/AKT/mTOR Pathway, and Suppress c-Myc Function in HCC Cells

**FIGURE 5:**
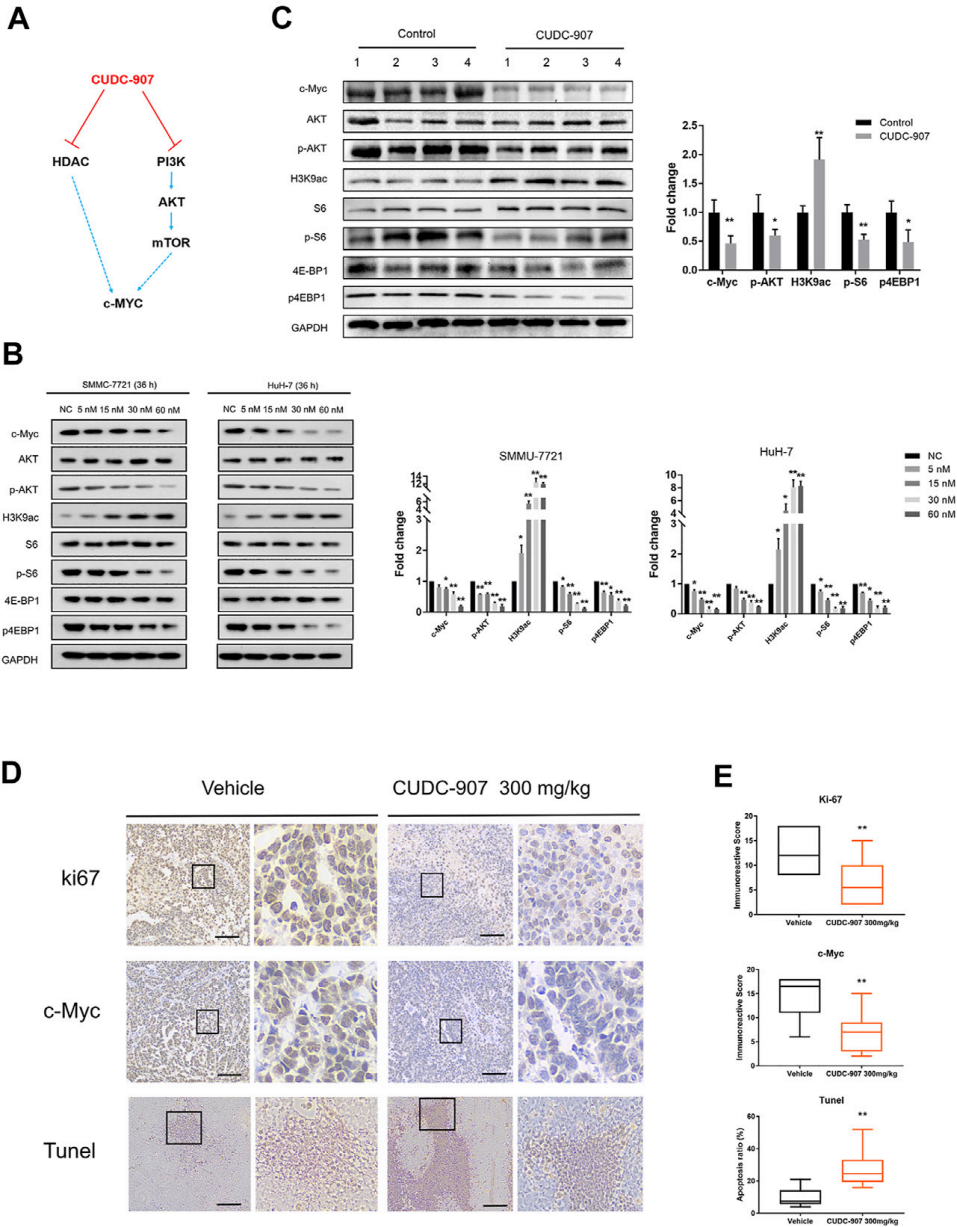
Western blotting of downstream regulated proteins of CUDC-907 and immunohistochemistry staining. **(A)** The expression of c-Myc is regulated by the HDAC and PI3K/AKT/mTOR pathway, and both can be inhibited by CUDC-907. **(B)** Expression levels of proteins [c-Myc, p-AKT, p-S6, p-4EBP1, and acetyl histone H3K9 (H3K9ac)] in HCC cell lines affected by CUDC-907 were detected by western blotting. GAPDH was set as the internal reference. **(C)** Expression levels of proteins (same as figure 5B) from xenografted tumor with or without CUDC-907 treatment were detected. **(D)** Immunohistochemistry staining. Representative images of Ki67, c-Myc, and TUNEL staining of xenografts treated with CUDC-907 or vehicle. **(E)** Quantification of immunohistochemistry staining was calculated by the immunohistochemistry score method. The percentage of apoptotic area in tumor slides of TUNEL staining was calculated by IMAGE J software. *p < 0.05, **p < 0.001 (result compared with Vehicle group).

CUDC-907 is a dual PI3K and HDAC inhibitor, and previous studies have demonstrated that HDAC inhibitors act synergistically with PI3K inhibitors to inhibit tumor growth in an MYC-dependent manner in different types of cancer (Fu et al., 2019; Simmons et al., 2017; Mondello et al., 2017; Pei et al., 2016) (Figure 5A). According to previous research on CUDC-907, the downregulation of c-Myc protein is an early event induced by CUDC-907 treatment, resulting in the growth restriction of c-Myc-driven cancers (Sun et al., 2017a). Importantly, HCC is a type of refractory cancer with predominant c-Myc overexpression (Xu et al., 2019), and the proliferation of HCC can be inhibited with a low concentration of CUDC-907, as suggested by the aforementioned results. To explore the mechanisms underlying this inhibition, we evaluated the expression of acetyl histone H3K9 (H3K9ac), the targets of PI3K and c-Myc in HCC cell lines and HCC xenograft tissue. The HCC cell lines SMMC-7721 and HuH-7 were treated with CUDC-907 at various concentrations for 36 h. Western blotting analysis revealed that CUDC-907 upregulated H3K9ac levels, reduced the expression of p-AKT, p-S6, and p4EBP1 (the targets of the PI3K/AKT/mTOR pathway), and suppressed the expression of c-Myc in HCC cell lines (Figure 5B). In addition, western blotting analysis in HCC xenograft tissue also confirmed the downregulation of c-Myc upon treatment with CUDC-907 (Figure 5C). Immunohistochemistry analysis of the xenograft HCC tumor showed that level of the cell proliferation biomarker Ki67 and that of c-Myc in the CUDC-907-treated group were downregulated compared to that in the untreated group (Figure 5D). Furthermore, the TUNEL assay showed that the apoptotic area in CUDC-907-treated tumor slides was larger than that in the untreated group (Figure 5D).

## Discussion

Hepatocellular carcinoma is the sixth most common malignancy and the fourth leading cause of cancer-related deaths worldwide (Llovet et al., 2016). In recent years, the first line targeted drugs for HCC are sorafenib and its alternative lenvatinib. However, the anticancer efficacy of sorafenib is unsatisfactory because it only prolongs the overall survival (OS) period by approximately 3 months compared with placebo. The efficacy of lenvatinib is better than that of sorafenib, but the improvement is limited (Liu et al., 2019). Thus, there is an urgent need to identify new potent anticancer drugs for HCC patients.

When it comes to drug screening for drug discovery and development, enormous amounts of money and time must be spent to obtain clinically approved drugs (Moffat et al., 2014). Even after the long and costly process to identify potential compounds, ∼80% of drugs failed during clinical trials and the most common reason for failure was lack of efficacy (Kondo and Inoue, 2019). Thus, choosing a more effective cancer model that has higher predictive power for anticancer drug screening can greatly improve the success rate of drug discovery and reduce the cost of the screening process. In recent years, the rapid development of 3D culture techniques has provided various pre-clinical models for drug screening.

Spheroid culture is a type of 3D culture technology that has been developed for nearly 3 decades (Landry et al., 1985). This technique is characterized by its low technical difficulty and cost, and it can well imitate the three-dimensional structure and metabolic function of tumors *in vivo* (Li et al., 1992; Dilworth et al., 2000). Thus, in a previous study, we developed a novel spheroid culture device for anti-tumor drug screening (Liao et al., 2019). Our novel 3D culture device is low cost, reusable, and suitable for various cells, including primary cancer cells, and is effective in controlling the size and shape of cultured spheroids, meaning that it is a proper tool in 3D drug screening. Therefore, in this study, we designed a set of schemes to screen the anti-HCC drugs by using our novel 3D culture device with the aims to find a potential anti-HCC molecular agent and the hope to establish an effective method for *in vitro* 3D drug screening.

First, we conducted a three-round 3D drug screening on HCC cell lines using our novel spheroid culture device. HuH-7 cells is one of the most commonly used cell lines for HCC study. Thus, we used Huh-7 cell for the first round of screening. After three rounds of screening, we found that CUDC-907 is a potential anti-HCC agent from 19 inhibitors. The results of the drug sensitivity test showed that CUDC-907 has a potent inhibitory effect on HCC cell lines at a low concentration and its efficacy is obviously better than that of the first-line anti-HCC drug sorafenib. Furthermore, functional experiments showed that CUDC-907 inhibited proliferation, caused G2/M arrest, and induced apoptosis in HCC cells. The above evidence suggests that CUDC-907 is a promising anti-HCC agent. CUDC-907, a dual PI3K/HDAC inhibitor, has been proposed to have therapeutic potential in many hematopoietic malignancies and solid cancers, such as chronic lymphocytic leukemia, acute myeloid leukemia, thyroid cancer, and pancreatic adenocarcinoma (Kotian et al., 2017; Chen et al., 2019b; Fu et al., 2019; Li et al., 2019). It has been recently granted Fast Track designation for the treatment of adults with relapsed or refractory diffuse large B-cell lymphoma by the US Food and Drug Administration (Li et al., 2019). Importantly, to the best of our knowledge, our research team was the first to determine that CUDC-907 has a potent inhibitory effect on HCC cells.

Based on the drug screening of HCC cell lines, in order to further verify the effect of CUDC-907 on HCC, we extracted primary HCC cells for the 3D drug-sensitive test. Primary cells from seven cases, including six cases of HCC and primary hepatocytes from one case, were successfully extracted and cultured. The results of the drug sensitivity test showed that the inhibitory efficacy of CUDC-907 on different patient-derived primary cells varied. The IC_50_ of CUDC-907 in different cases of primary HCC ranged from 1.7 to 130 nM (0.86–65.76 ng/ml), which was much lower than the IC_50_ of sorafenib, and the killing ability of CUDC-907 on primary hepatocyte was weak. These results suggest that the *in vitro* inhibitory effect of CUDC-907 on primary HCC cells is stronger than that of sorafenib, and that there are differences in drug responses among different individuals. Indeed, HCC is a highly heterogeneous disease in terms of its molecular profiles. The genome variation and expression differ among different individuals and in different parts of tumors; this feature is one of the most important reasons for the various drug responses (Gao et al., 2017). The wide range of IC_50_ of CUDC-907 on primary HCC cells indicates that different patients are potentially resistant to CUDC-907, which highlights the importance of a valid preclinical HCC model and individualized treatment. It is also meaningful to research the mechanism, which may provide guide for the individualized precision treatment. CUDC-907 targets PI3K and HDAC. We examined four common PI3K mutations (which can highly activate the p-AKT pathway) in five HCC samples by the Sanger method of DNA sequencing, and did not detect any PI3K mutations in these samples (Supplementary Figure S1B). Then, the expression levels of H3K9ac, AKT, p-AKT, and c-Myc in these five samples were measured by western blot (Supplementary Figure S1C). Univariate linear regression analysis between IC_50_ and the expression of H3K9ac, p-AKT, and c-Myc revealed that there was no significant correlation between them (Supplementary Figure S1D). Considering the small sample size, this still needs to be investigated and verified in the future.

Although a previous report pointed out that the peak plasma concentration of CUDC-907 was around 5 ng/ml, the concentration of CUDC-907 in tumor samples could reach approximately 69.5 ng/g (Younes et al., 2016b). Whether CUDC-907 is effective for HCC treatment *in vivo* remains unknown. We next conducted an animal study to further prove the effect of CUDC-907 *in vivo*. Initially, we planned to establish patient-derived xenograft (PDX) mouse models by subcutaneous engraftment of tumor species. However, this experiment failed as the engrafted tissues did not grow *in vivo*. For substitution, we established an SMMC-7721 HCC xenograft. The results of animal experiments suggested that CUDC-907 could significantly suppress the growth of HCC tumors *in vivo*. This evidence suggests that CUDC-907 might be a potential anti-HCC agent, and our results imply that the 3D *in vitro* drug screening method we used in this study may be a valuable method for drug screening and discovery. However, as mentioned above, the oral absorption of CUDC-907 is still low (Younes et al., 2016b). Therefore, further improvement of its oral bioavailability may be needed to enhance its anticancer effect.

Previous reports have shown that CUDC-907 is a dual-acting inhibitor of PI3K and HDAC, and exhibits predominant anticancer effects in c-Myc-driven tumors (Ferreira et al., 2016; Sun et al., 2017b). The c-Myc oncoprotein is an essential transcription factor that regulates the expression of many genes involved in cell growth, proliferation, and metabolic pathways (Farrell and Sears, 2014). The deregulation and enhancement of c-Myc play an essential role in the carcinogenesis and progression of virous cancer, including HCC (Srivastava et al., 2015). In order to understand the anti-HCC effect of CUDC-907, we investigated the expression levels of possible target molecules of CUDC-907. We used western blotting to investigate the impact of CUDC-907 on HDAC, PI3K, and c-Myc in HCC cells. Western blotting of HCC cell lines and xenograft tissues showed that CUDC-907 can inhibit the HDAC and PI3K/AKT/mTOR pathway and suppress the expression of c-Myc. It is worth mentioning that the PI3K/AKT/mTOR pathway is also a central regulator of various oncogenic processes including cell growth, proliferation, metabolism, and angiogenesis in HCC (Ashworth and Wu, 2014). Thus, the dual inhibition of c-Myc and PI3K/AKT/mTOR may be the main reason that contributes to the potent effect of CUDC-907 on HCC.

In summary, the 3D spheroid-based drug screening we established in this experiment presents a valuable application for novel anti-tumor drug screening and personalized treatment. The data in this study suggested that CUDC-907 might be a promising drug for HCC treatment. However, this study has some limitations. Firstly, testing with a positive drug (such as sorafenib) was not included in animal experiments. Therefore, it is not clear whether the anticancer effect of CUDC-907 is superior to that of sorafenib *in vivo*. Secondly, PDX studies and clinical research are still needed to validate its efficacy in HCC treatment.

## Data Availability

The raw data supporting the conclusion of this article will be made available by the authors, without undue reservation.
