# Supplementary material for: Therapeutic Potential of CUDC-907 (Fimepinostat) for Hepatocarcinoma Treatment Revealed by Tumor Spheroids-Based Drug Screening
Source: Front Pharmacol. 2021 Oct 29;12:658197. doi: 10.3389/fphar.2021.658197 (PMC8585736; doi:10.3389/fphar.2021.658197)
Supplement: Supplementary file 1 [file Image1.pdf]

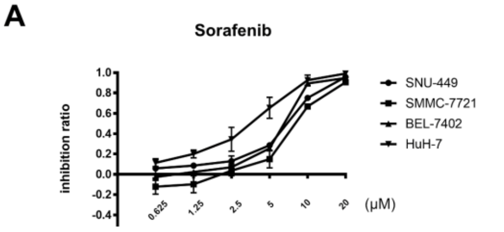

B

Sample	rs121913279 A>G,T	rs121913273 G>A,C	rs121913275 G>A,C,T	rs104886003 G>A,C
HCC-1	A	G	G	G
HCC-2	A	G	G	G
HCC-3	A	G	G	G
HCC-4	A	G	G	G
HCC-5	A	G	G	G

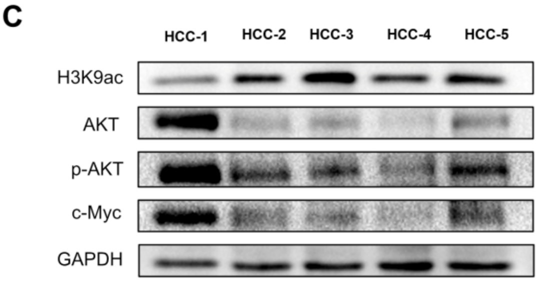

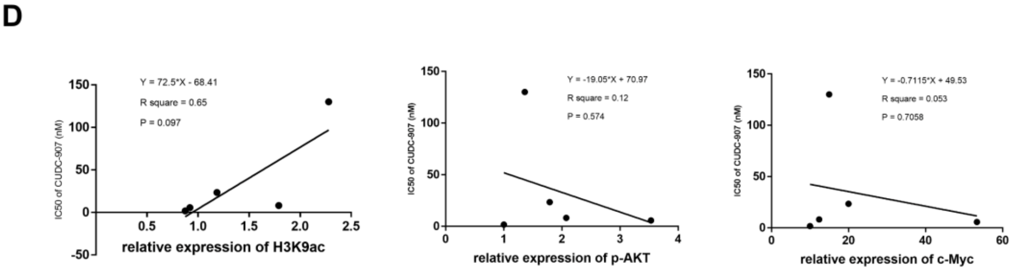

Supplementary figure 1 | (A) Inhibitory curve of sorafenib on HCC cell lines. HCC cell lines were cultured in our novel spheroid culture device and treated with sorafenib of different concentrations for 3 days. **(B)** Four common PI3K mutations were examined in 5 HCC samples by the Sanger method of DNA sequencing. The result shown that no mutation was detected. **(C)** Expression levels of proteins including H3K9ac, AKT, p-AKT, and c-Myc in 5 HCC samples were detected by western blotting. GAPDH was set as the internal reference. **(D)** The univariate linear regression analysis between IC50 and the levels of H3K9ac, p-AKT, and c-Myc in five HCC samples was calculated with SPSS 20.0 software.
